# Synthesis and Biological Evaluation of Novel ^99m^Tc-Labelled Bisphosphonates as Superior Bone Imaging Agents

**DOI:** 10.3390/molecules16086165

**Published:** 2011-07-25

**Authors:** Ling Qiu, Wen Cheng, Jianguo Lin, Shineng Luo, Li Xue, Jing Pan

**Affiliations:** Key Laboratory of Nuclear Medicine, Ministry of Health, Jiangsu Key Laboratory of Molecular Nuclear Medicine, Jiangsu Institute of Nuclear Medicine, Wuxi 214063, China

**Keywords:** zoledronic acid derivative, ^99m^Tc-labelled diphosphonates, *in vitro* stability, biodistribution, bone imaging agent

## Abstract

A series of novel zoledronic acid (ZL) derivatives 1-hydroxy-3-(2-methyl-1*H*-imidazol-1-yl)propane-1,1-diyldiphosphonic acid (MIPrDP), 1-hydroxy-4-(2-methyl-1*H*-imidazol-1-yl)butane-1,1-diyldiphosphonic acid (MIBDP), and 1-hydroxy-5-(2-methyl-1*H*-imidazol-1-yl)pentane-1,1-diyldiphosphonic acid (MIPeDP) were prepared and successfully labeled with ^99m^Tc in high labeling yields. The *in vitro* stability and *in vivo* biodistribution of ^99m^Tc-MIPrDP, ^99m^Tc-MIBDP and ^99m^Tc-MIPeDP were investigated and compared. The biodistribution studies indicate that the radiotracer ^99m^Tc-MIPrDP has highly selective uptake in the skeletal system and rapid clearance from soft tissues. The present findings indicate that ^99m^Tc-MIPrDP holds great potential for use in bone imaging.

## 1. Introduction

Technetium-99m, with its excellent physical characteristics and easy availability from a generator, has become the most important nuclide for organ imaging in nuclear medicine. Since Fleisch *et al*. described that bisphosphonates had high affinity for bone mineral in 1968, several ^99m^Tc-labeled phosphate compounds have been developed for skeletal imaging, including pyrophosphate [1], polyphosphates [2,3], and diphosphonates [4,5]. 

Complexes of technetium-99m labeled with methylenediphosphonate (^99m^Tc-MDP) and hydroxymethylenediphosphonate (^99m^Tc-HMDP) have been widely used, both experimentally and clinically, for detection of skeletal metastases and other focal bone lesions [4,6,7]. However, an interval of 2–6 h is needed between injecting these agents into the patient and bone scanning [8]. Shorting this interval would lessen the burden on patients in terms of the total length of the examination. To enable imaging at an earlier time after injection, a radiopharmaceutical with higher affinity for bone, larger ratio of bone-to-soft tissue uptake and more rapid clearance from blood is required, accordingly [9]. Consequently, the nature of ligands (diphosphonic acid) is a key factor to determine the advantages of the radiopharmaceuticals.

Diphosphonates have a fundamental P–C–P backbone structure, and have high affinity for bone mineral. ZL [1-hydroxy-2-(imidazol-1-yl)-ethylidene-1,1-bisphosphonic acid], one kind of the typical third-generation DPs, is currently the most potent bisphosphonate. In preclinical models of bone resorption, for example, ZL is at least 100 times more potent than clodronate and pamidronate, and it is at least 1,000 times more potent than etidronate [10]. 

In the previous work of our group, a series of technetium-99m labeled DPs with the alkyl substituent in the imidazole ring of ZL have been prepared and their *in vivo* biological properties were systematically investigated [11,12,13,14]. However, to the best of our knowledge, extension and optimization of the linker chain between the imidazolyl and geminal bisphosphonate group to develop novel bone imaging agent has been largely unexplored.

**Figure 1:**
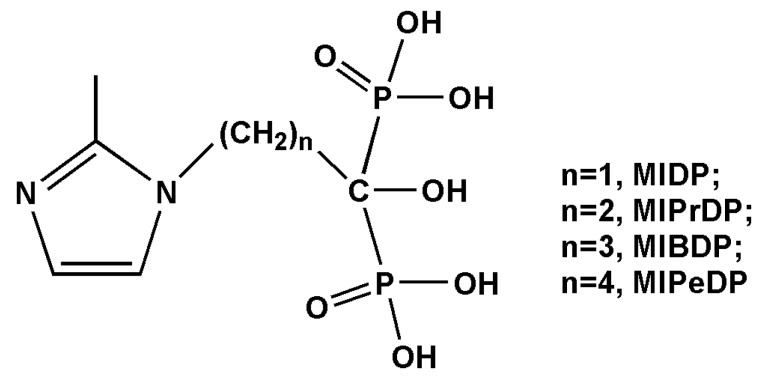
Structures of MIDP and its derivatives.

Based on our primary studies, we found that optimization of imidazolyl ring or extension of the linker chain between the imidazolyl and geminal bisphosphonate group in the ZL to be labeled with technetium-99m can bring significant influence on the biological properties of the bone resorption and the clearance from blood and soft tissues [15,16]. For the purpose of developing novel bone imaging agent with excellent biological properties, we continue to extend the number of the methylene chain between the methylimidazolyl and geminal bisphosphonate group in the diphosphonate acid (see Figure 1). In this work, a series of novel ^99m^Tc-labeled bisphosphonates were prepared and reported, *i.e*., ^99m^Tc-MIPrDP, ^99m^Tc-MIBDP and ^99m^Tc-MIPeDP. Their *in vitro* stability and *in vivo* biodistribution were also studied. 

## 2. Results and Discussion

### 2.1. Chemistry and Radiolabeling

MIPrDP, MIBDP and MIPeDP were synthesized by three step reactions from the starting material 2-methylimidazole. The target compounds were identified by elemental analysis, MS and ^1^H-NMR, and the results agree well with the expected chemical structures. ^99m^Tc-MIPrDP, ^99m^Tc-MIBDP and ^99m^Tc-MIPeDP were labeled with ^99m^TcO_4_^−^ by reduction with stannous chloride. For TLC analysis, with the H_2_O system, the R*_f_* values of Na^99m^TcO_4_ and **5a-5c** were about 0.9-1, while ^99m^Tc-colloidal impurities remain at 0-0.1. With the acetone system, the ^99m^Tc-colloidal impurities and **5a-5c** remain at the origin and Na^99m^TcO_4_ moves with the solvent front.

**Figure 2:**
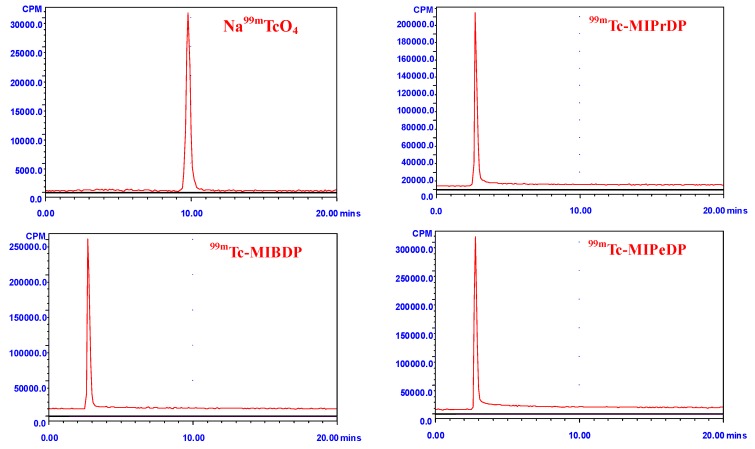
HPLC chromatograms (**5a** rt = 2.8 min, **5b** rt = 2.9 min, **5c** rt = 2.9 min and ^99m^TcO4^−^ rt = 9.8 min).

HPLC analysis revealed the formation of free technetium (Na^99m^TcO_4_) that was eluted at a retention time of 9.8 min, whereas **5a-5c** eluted at a retention time of 2.8 ± 0.1 min (see Figure 2). The identical retention times revealed the structural analogy of these ^99m^Tc-DPs [9]. For each radiolabeled complex, the single peak in the HPLC-chromatogram clearly shows the formation of only one complex and excludes the possibility of residual Na^99m^TcO_4_ or other components [17]. That is, the chelation of technetium with the bisphosphonates moiety is unique and complete.

According the TLC and HPLC analysis, the radiochemical purities of these ^99m^Tc-DPs were all greater than 97%. This labeling method also meets the clinical requirement of other ^99m^Tc-labeled bisphosphonates such as ^99m^Tc-MDP. The radiolabeled compounds were used immediately after the formulation for both *in vitro* and *in vivo* studies. 

### 2.2. *In Vitro* Stability and Octanol-Water Partition Coefficient

The *in vitro* stabilities of ^99m^Tc-MIPrDP, ^99m^Tc-MIBDP and ^99m^Tc-MIPeDP were studied in PBS (pH = 7.4) for different time intervals (1, 2, 3, 4, 5, 6 h) at 37 °C. The stability was presented as RCP on the basis of the HPLC analysis. After 6 h of incubation, more than 97% of ^99m^Tc-MIPrDP, ^99m^Tc-MIBDP and ^99m^Tc-MIPeDP remained intact in the PBS. The results indicated that the labeling efficiency of these complexes was high and their stability duration was long enough to allow further biodistribution and imaging studies. 

The octanol-water partition coefficients (log*P*) for ^99m^Tc-MIPrDP, ^99m^Tc-MIBDP and ^99m^Tc-MIPeDP were −1.89, −1.93, −2.10 and −1.71, −1.77, −1.89 in PBS at two different pH values of 7.0 and 7.4 respectively (see Table 1), which demonstrated that log*P* decreased from ^99m^Tc-MIPrDP to ^99m^Tc-MIPeDP and the liposolubility at pH = 7.4 was higher than that at pH = 7.0. From ^99m^Tc-MIPrDP to ^99m^Tc-MIPeDP, the longer the carbon chain, the smaller the log *P*. As well known, the log *P* value is a very useful parameter that can be used to understand the behavior of a drug and predict its distribution in the organism in combination with other parameters [18]. The different lipotropy among three technetium-99m labeled zoledronic acid derivatives may be attributed to the change in the linker chain between the methylimidazolyl and the geminal bisphosphonate group.

### 2.3. Plasma Protein Binding

The percentage of protein binding was 11.2%, 12.4%, and 14.7% for ^99m^Tc-MIPrDP, ^99m^Tc-MIBDP and ^99m^Tc-MIPeDP, respectively. As known, different protein binding rate has significant influence on the bone uptake of the tracer agent [19]. Here, with the extension of carbon chain, the plasma protein binding increases and the maximum bone uptake decreases gradually. The structural difference further affects the blood clearance of the radiolabeled complexes [20], which will be discussed in detail in the next blood kinetics studies.

### 2.4. Blood Kinetics Studies

**Figure 3:**
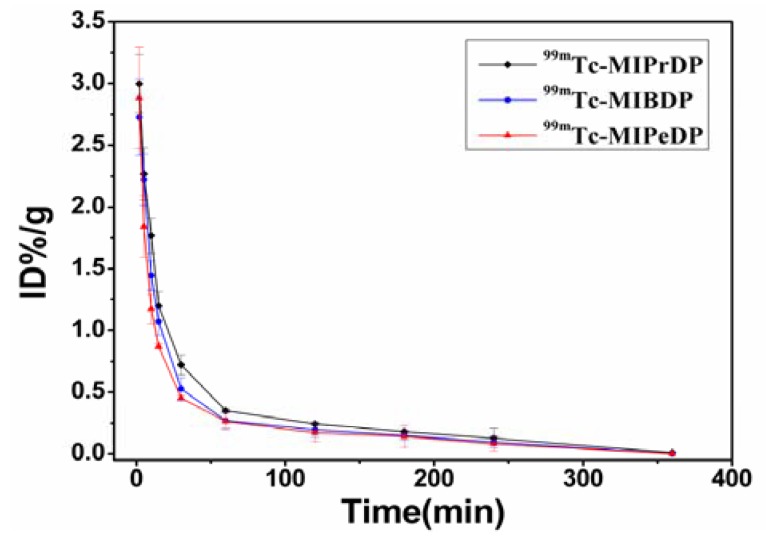
Pharmacokinetic curves in the mice for ^99m^Tc-MIPrDP, ^99m^Tc-MIBDP and ^99m^Tc-MIPeDP (n = 5, mean ± SD).

Pharmacokinetic parameters were listed in Table 2. Figure 3 shows the blood clearance of **5a-5c** in the mice 6 h post injection. Pharmacokinetics of **5a-5c** comply with the two-compartment model with the pharmacokinetic equations of C = 2.66e^−0.16t^ + 0.90e^−0.01t^, C = 2.53e^−0.056t^ + 0.282e^−0.0034t^ and C = 2.03e^−0.061t^ + 0.736e^−0.0067t^, respectively. The values of CL were 2.58, 2.90 and 3.84 and the AUC were 142.9, 127.4, and 96.2 for ^99m^Tc-MIPrDP, ^99m^Tc-MIBDP and ^99m^Tc-MIPeDP, respectively.

In the early phase, the blood clearance of ^99m^Tc-MIPrDP was slower than ^99m^Tc-MIBDP and ^99m^Tc-MIPeDP. After 2 h, the radioactivity concentration of three tracer agents in blood reaches an equilibrium which coincides with the pharmacokinetic parameters CL, AUC and the pharmacokinetic curves. Ranging from 5 to 10 min, the bone uptake increases with the increased carbon chain. That is, the uptake of ^99m^Tc-MIPeDP in bone increases faster than ^99m^Tc-MIPrDP and ^99m^Tc-MIBDP, which consists with the variation of K_12_. It was concluded that ^99m^Tc-MIPeDP can be absorbed quickly and eliminated from the blood rapidly. In this study, the blood clearance (see Figure 3) of ^99m^Tc-MIPrDP, ^99m^Tc-MIBDP and ^99m^Tc-MIPeDP were very fast, due to low protein binding.

### 2.5. Biodistribution Studies

Biodistributions of **5a-5c** were determined in ICR mice, and the data is shown in Table 3 as the percentage administered activity (injected dose) per gram of tissue (%ID/g). Although ZL has been known to possess low toxicity and can be used therapeutically at a high dose [21,22], the toxicity profile of its derivatives are uncertain. Therefore, after the complexes ^99m^Tc-DPs (1.85 MBq) in a volume of 0.2 mL were given to the normal mice via intravenous injection respectively, the mice were watched carefully for any sign of adverse reaction. As expected, the mice showed no signs of toxicity through the overall study period. 

Inspecting Table 3, one can observe that **5a-5c** are mainly accumulated in the bone, kidneys, and liver. The uptake of **5a-5c** in bone increased steadily from 5 to 60 min post injection. At 2 h post injection, the bone uptake was 19.6 ± 0.87, 11.2 ± 0.13 and 17.6 ± 0.42 %ID/g for ^99m^Tc-MIPrDP, ^99m^Tc-MIBDP and ^99m^Tc-MIPeDP, respectively. From previous work of our group [16], it was found that the bone uptake increases with the increasing carbon chain between the imidazolyl and geminal bisphosphonate group in ZL (*i.e*., from ^99m^Tc-ZL to ^99m^Tc-IPrDP). However, in the present work the bone uptake of ^99m^Tc-MIPeDP was smaller than those of ^99m^Tc-MIPrDP and ^99m^Tc-MIBDP (see Table 3). This indicates that limitless extension of the carbon chain is not always beneficial to improve the bone uptake. Furthermore, the bone uptake efficiency of these radiotracers was compared. For example, after injection of ^99m^Tc-MIPrDP, the bone uptake was up to 6.43 %ID/g at 5 min and it increased continuously to a maximum of 19.6 %ID/g at 120 min, which was larger than the corresponding values of ^99m^Tc-IPrDP (5.37 and 11.14 %ID/g respectively) [16]. This implied that the bone uptake efficiency of the former with a methyl in the imidazole ring is larger than the latter with no substituent in the imidazole ring. Considering that ^99m^Tc-IPrDP is better than ^99m^Tc-MDP and ^99m^Tc-ZL in the bone imaging, it is therefore concluded that ^99m^Tc-MIPrDP is the best one among these bone scanning agents.

**Figure 4:**
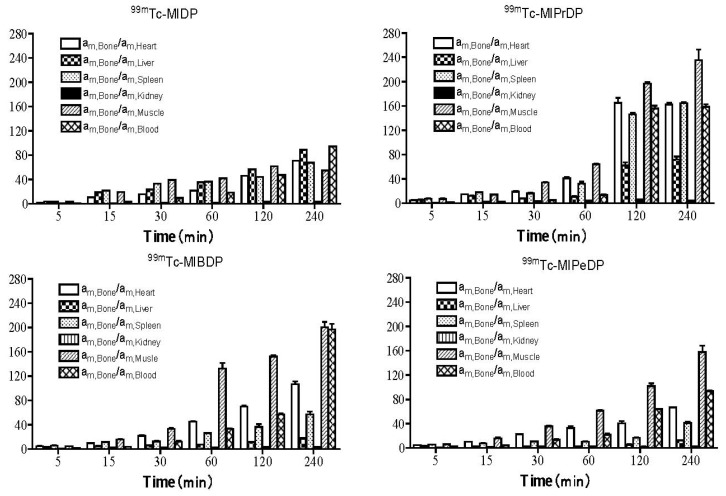
Bone to tissue uptake ratio in mice at different time post injection of ^99m^Tc-MIDP and **5a-5c**.

Compounds **5a-5c** also showed predominant kidney and liver uptake, indicating that they were not only excreted through the kidneys, but also absorbed and eliminated by the liver. It was noted that the uptake of ^99m^Tc-MIBDP and ^99m^Tc-MIPeDP in liver, spleen and kidneys were larger than those of ^99m^Tc-MIPrDP. For instance, the kidneys and liver uptake of **5a-5c** at 2 h post injection were 3.21 ± 0.50, 4.45 ± 0.22 and 4.80 ± 0.14 %ID/g for kidneys and 0.32 ± 0.05, 1.14 ± 0.04 and 2.25 ± 0.24 %ID/g for liver, respectively. The uptake ratios of bone to liver and kidneys for ^99m^Tc-MIPrDP were 61.25 ± 1.89 and 6.10 ± 0.78 respectively, while those of ^99m^Tc-MIBDP and ^99m^Tc-MIPeDP were 9.81 ± 1.23, 2.51 ± 0.69 and 7.81 ± 0.74, 3.6 ± 1.21 (see Figure 4). Thus, we can see that from ^99m^Tc-MIPrDP to ^99m^Tc-MIPeDP with the extension of the linker chain between the methylimidazolyl and geminal bisphosphonates group, the former exhibits a significant advantage in the clearance from soft tissues and it can better satisfy the clinical requirements of a good bone imaging agent.

Radioactivity levels of **5a-5c** in the blood were 5.00 ± 0.02, 3.79 ± 0.07 and 3.40 ± 0.14 %ID/g at 5 min post injection, respectively, which followed by a rapid clearance. At 4 h post injection, the radioactivity in blood was only 0.08, 0.09 and 0.12 %ID/g for **5a-5c** respectively. This indicates that all these radiotracers can eliminate quickly from the blood, which agrees well with the pharmacokinetics studies. 

For better understanding the biodistributions and metabolism of **5a-5c** *in vivo*, we further compared them with the radiotracer ^99m^Tc-MIDP (1-hydroxy-2-(2-methyl-1*H*-imidazolyl)ethane-1,1-diyl diphosphonic acid) [11]. The biodistribution data also show that ^99m^Tc-MIDP has high kidney uptake, indicating that it is excreted quickly through the kidneys. Comparison of the uptake ratios of bone to soft tissue (including heart, liver, spleen, muscle and kidneys) among these homologues shows that these uptake ratios of ^99m^Tc-MIDP are all very small while those of ^99m^Tc-MIPrDP are all larger than others after 120 min post injection (see Figure 4). This showed ^99m^Tc-MIPrDP was not only superior to its parent complex ^99m^Tc-MIDP but also superior to ^99m^Tc-IPrDP judged from the biodistribution studies. In summary, the radiotracer ^99m^Tc-MIPrDP better meets the clinical requirement of bone imaging agent with higher bone uptake and lower background among this kind of homologues. 

## 3. Experimental 

### 3.1. General

All analytical chemical reagents employed were purchased from commercial sources and used without further purification. Na^99m^TcO_4_ was supplied by the Affiliated Jiangyuan Hospital of the Jiangsu Institute of Nuclear Medicine. All melting points were measured on a Yanaco MP-500 melting point apparatus (Shimadzu, Japan). Elemental analysis was carried out using an Elementar Vario EL III analyzer. Electron spray ion (ESI) mass spectra were determined using a Waters Platform ZMD4000 LC/MS (Waters, U.S.A.). Nuclear magnetic resonance (NMR) spectra were obtained on a Bruker DRX-500 spectrometer (Bruker, Germany), and the chemical shift value was given relative to the internal tetramethylsilane (TMS). Xinhua chromatography paper (Shanghai, China) was used for thin layer chromatography (TLC). A Packard-multi-prias γ Counter (Perkins Elmer, U.S.A.) was used. High performance liquid chromatography (HPLC) analysis was performed on a Waters 600-type high-performance liquid chromatography (Waters, U.S.A.) equipped with a dural k absorbance detector (Waters 2487), binary HPLC pump (Waters 1525) and Cd (Te) detector equipped with a flow scintillation analyzer (Perkin Elmer). Normal Institute of Cancer Research (ICR) mice (weighting 18~20 g) were supplied by the Shanghai SLAC Laboratory Animal CO., Ltd. (Shanghai, China). The animal experiment was approved by the Animal Care and Ethics Committee of the Jiangsu Institute of Nuclear Medicine.

### 3.2. Syntheses of Diphosphonic Acids

**Scheme 1:**
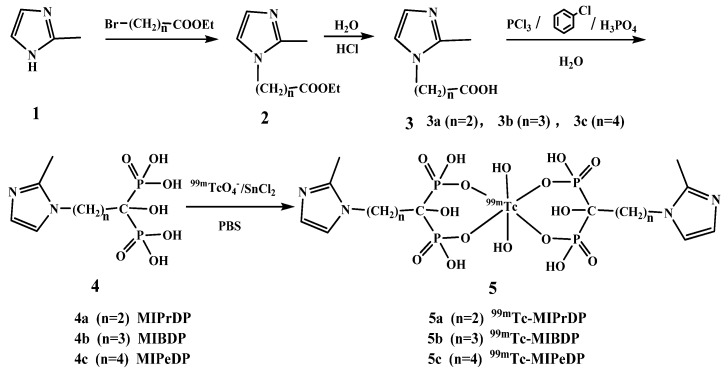
Syntheses of ^99m^Tc-MIPrDP, ^99m^Tc-MIBDP and ^99m^Tc-MIPeDP.

Three ^99m^Tc-diphosphonates were synthesized according to the procedure outlined in Scheme 1 as described previously [16,23].

#### 3.2.1. General Procedure for the Preparation of Compounds **3a-3c**

2-Methylimidazole (**1**, 8.2 g, 0.1 mol) was dissolved in CH_2_Cl_2_ (75 mL). Then KOH (8.4 g, 0.15 mol), K_2_CO_3_ (13.8 g, 0.0835 mol) and tetrabutylammonium bromide (0.7 g, 0.002 mol) were added and stirred at room temperature for 30 min. The solution was treated dropwise with the corresponding ethyl bromoacetate homolog (0.1 mol) at room temperature. The mixture was heated to reflux for 7 h. The inorganic salt was removed by filtration and the filtrate was washed with saturated NaCl solution (30 mL × 4). Finally, the organic layer was evaporated under vacuum to give crude compound **2** as a brown gum, which was used without any further purification for the next step. Water (90 mL) and HCl (12 mol/L, 10 mL) were successively added to compound **2**, and the mixture was refluxed 8 h, the solution was concentrated and recrystallized from isopropanol to give the white crystalline product **3**.

*3-(2-Methyl-1H-imidazol-1-yl)propanoic acid* (**3a**): Yield: 34%. mp 125-128 °C; ESI-MS, *m/z* (%): 153 (100) = M-H^+^.

*4-(2-Methyl-1H-imidazol-1-yl)butanoic acid* (**3b**): Yield: 42%. mp 120-122 °C; ESI-MS, *m/z* (%): 167 (100) = M-H^+^.

*5-(2-Methyl-1H-imidazol-1-yl)pentanoic acid* (**3c**): Yield: 30%. mp 123-125 °C; ESI-MS, *m/z* (%): 181 (100) = M-H^+^.

#### 3.2.2. General Procedure for the Preparation of Compound **4**

Compound **3** (20 mmol) was dissolved in chlorobenzene (25 mL) and heated to 120 °C for 30 min, then phosphoric acid (85%, 4.2 mL) was added. The solution was treated dropwise with phosphorus trichloride (7.6 mL), and kept at 120 °C for 4 h. The chlorobenzene was decanted. Then, the yellow residue was redissolved in HCl (9 mol/L, 20 mL) and heated to reflux for 5 h. The mixture was treated with charcoal before filtration and concentration. Finally, the crude product was recrystallized from ethanol to give the white crystalline product **4**.

*1-Hydroxy-3-(2-methyl-1H-imidazol-1-yl)propane-1,1-diyldiphosphonic acid* (**4a**): Yield: 65%. mp 183-186 °C; ^1^H-NMR (400 MHz, D_2_O): δ7.332 (d, 1H, CH-ring), 7.208 (d,1H, CH-ring), 4.378 (t, 2H, N-CH_2_), 2. 557 (s, 1H, ring-CH_3_), 2.419 (q, 2H, OH-C-CH_2_); ^13^C-NMR (125 MHz, D_2_O) δ: 144.216, 121.349, 117.808 (3C, *C*-ring), 71.896 (*C*-OH), 43.167 (N-*C*H_2_), 33.599 (*C*H_2_), 9.943 (-*C*H_3_); ESI-MS, *m/z* (%): 299 (100) = M-H^+^. Anal. calcd for C_7_H_14_N_2_O_7_P_2_ (%): C, 28.01; H, 4.70; N, 9.33; Found (%): C, 28.22; H, 4.82; N, 9.51.

*1-Hydroxy-4-(2-methyl-1H-imidazol-1-yl)butane-1,1-diyldiphosphonic acid* (**4b**): Yield: 58%. mp 142-145 °C; ^1^H-NMR (400 MHz, D_2_O): δ7.635 (d, 1H, CH-ring), 7.547 (d, 1H, CH-ring), 4.115 (t, 2H, N-CH_2_), 2.592 (s, 1H, ring-CH_3_), 2.320 (q, 2H, OH-C-CH_2_), 2.004 (m, 2H, CH_2_-CH_2_-CH_2_); ^13^C-NMR (125 MHz, D_2_O) δ: 146.160, 124.981, 120.525 (3C, *C*-ring), 74.692 (*C*-OH), 46.669 (N-*C*H_2_), 31.901, 25.431 (2C, *C*H_2_), 11.813 (-*C*H_3_); ESI-MS, *m/z* (%): 231 (100) = M^+^-83. Anal. calcd for C_8_H_16_N_2_O_7_P_2_ (%): C, 30.61; H, 5.09; N, 8.92; Found (%): C, 30.72; H, 5.24; N, 9.01.

*1-Hydroxy-5-(2-methyl-1H-imidazol-1-yl)pentane-1,1-diyldiphosphonic acid* (**4c**): Yield: 70%. mp 212-215 °C; ^1^H-NMR (400 MHz, D_2_O): δ7.295 (d, 1H, CH-ring), 7.208 (d, 1H, CH-ring), 4.072 (t, 2H, N-CH_2_), 2.533 (s, 1H, ring-CH_3_), 1.943 (q, 2H, OH-C-CH_2_), 1.824 (m, 2H, CH_2_-CH_2_-COH), 1.606 (m, 2H, ring-CH_2_CH_2_); ^13^C-NMR (125 MHz, D_2_O) δ: 144.388, 121.271, 119.475 (3C, *C*-ring), 74.209 (*C*-OH), 46.965 (N-*C*H_2_), 33.678, 30.169, 21.143(3C, *C*H_2_), 10.396 (-*C*H_3_); ESI-MS, *m/z* (%): 245 (100) = M^+^-83. Anal. calcd for C_9_H_18_N_2_O_7_P_2_ (%): C, 32.93; H, 5.49; N, 8.54; Found (%): C,33.04; H, 5.52; N, 8.39.

### 3.3. Radiochemical Syntheses of ***5a-5c***

To a 10 mL vial, aqueous solution of **4a-4c** (100 μL, 0.25 g DPs dissolved in 5.0 mL 0.2 mol/L sodium hydroxide solution), freshly prepared solution of stannous chloride dehydrate (100 μL, 10 mg SnCl_2_·2H_2_O dissolved in 10.0 mL 0.5 mol/L HCl), and 74.0 MBq freshly eluated Na^99m^TcO_4_ were added. The reaction solution was adjusted to pH = 6.0 by adding 0.2 mol/L phosphate buffered solution (PBS) and diluted to 2 mL. It was vortexed adequately and reacted at 70 °C for 30 min.

### 3.4. Quality Control of **5a-5c**

The radiochemical purity (RCP) and radiolabeling yield (RLY) of **5a-5c** was determined by TLC and HPLC.

#### 3.4.1. TLC

About 3 μL **5a-5c** solutions were spotted with a microcap at 1.5 cm from the bottom of paper strips. The paper strips were placed in two developing agents, acetone and distilled water. With the distilled water, ^99m^Tc-colloidal impurities remain at the bottom on the paper strip, while Na^99m^TcO_4_ and **5a-5c** both migrate with the solvent front. With the acetone, the ^99m^Tc-colloidal impurities and **5a-5c** remain at the origin and Na^99m^TcO_4_ moves with the solvent front. The strips were cut into pieces of 1 cm and the activity of these pieces was counted to determine the RCP value by a well-type γ counter.

#### 3.4.2. HPLC

The RCP of **5a-5c** were determined a Waters 600-type high-performance liquid chromatography. The sample was passed through a millipore filter carefully and injected into the HPLC column (SinoChrom ODS-BP, PN: E2117215-080108, 4.6 mm × 250 mm × 10 μm, DaLian, China). The absorbance was measured on the UV detector at 210 nm. Radioanalysis of the labeled compound was conducted using a Cd (Te) detector. The flow rate was adjusted to 0.9 mL·min^−1^ and the isocratic mobile phase was 70% H_2_O and 30% CH_3_CN. 

### 3.5. *In Vitro* Stability of **5a-5c**

The *in vitro* stabilities of ^99m^Tc-MIPrDP, ^99m^Tc-MIBDP and ^99m^Tc-MIPeDP were studied in PBS (pH = 7.4) after different interval (1, 2, 3, 4, 5 and 6 h) at physiological temperature of 37 °C. The RCP values were evaluated by HPLC at different time points to determine if they were stable *in vitro*.

### 3.6. Octanol-Water Partition Coefficients of ***5a-5c***

Octanol–water partition coefficients were determined for **5a-5c** at two different pH values of 7.0 and 7.4 by measuring the distribution of radiolabeled compounds in *n*-octanol and PBS, respectively. A sample of radiolabeled compound **5a-5c** (100 μL) was diluted with PBS (900 μL). Then, it was mixed with *n*-octanol (1 mL) and vortexed at room temperature for 2 min. The mixture was further centrifuged at 4,000 rpm for 5 min to ensure complete separation of layers. Both layers were collected and the radioactivity counts from 100 μL aliquots of both the organic and aqueous phases were measured with a γ counter. Log *P* values were calculated using the formula of log *P* = log (CPM of octanol/CPM of water).

### 3.7. Plasma protein Binding Assay

The radiolabled compounds (100 μL, 37 KBq) were mixed with the human plasma (100 μL) in the centrifuge tube. After the mixture was incubated at 37 °C for 2 h, the plasma protein was precipitated by adding trichloroacetic acid (1 mL, 250 g/L) to the mixture. The supernatant and precipitate were separated by centrifugation at 2,000 rpm for 5 min. The radioactivities of both phases were measured separately. The above experimental procedure was repeated three times. The percentage of protein binding was determined by the following equation: plasma binding % = [plasma (CPM) − supernatant (CPM)/plasma (CPM)] × 100 = [precipitate (CPM)/plasma (CPM)] × 100%

### 3.8. *In Vivo* Distribution of ***5a-5c***

Thirty mice were randomly divided into six groups and injected via the tail vein with the test agent (^99m^Tc-MIPrDP, ^99m^Tc-MIBDP and ^99m^Tc-MIPeDP) in the volume of 0.2 mL and activity of approximately 7.4 MBq. Groups of mice were sacrificed by decapitation at 5, 15, 30, 60, 120 and 240 min after injection. Tissue samples of interest were removed and weighed, as well as 200 μL blood were taken from carotid artery. The activity for each sample was determined by a γ counter. Distribution of the radioactivity in different tissues and organs was calculated and expressed as the %ID/g, where:

$$\%\mathrm{ID}/g=\frac{\mathrm{organ}\;\mathrm{CPM}}{\mathrm{organ}\;\mathrm{weight}\times \mathrm{injected}\;\mathrm{CPM}}\times 100\%$$

### 3.9. Blood Kinetics Studies

For pharmacokinetics study, **5a-5c** (7.4 MBq, 0.2 mL) was administered to the mice via intravenous injection in the tail vein respectively. A series of blood samples (20 μL) were collected in the microcap tube by nicking the tail with a needle at 2, 5, 10, 15, 30, 60, 120, 180, and 240 min after injecting **5a-5c**. The radioactivity of each blood sample was counted and expressed as %ID/g. Pharmacokinetic parameters were analyzed by the program 3P97, and the radioactivity can be expressed as a function of time with the following equation C = Ae^−αt^ + Be^−βt^.

## 4. Conclusions

A series of novel ZL derivatives MIPrDP, MIBDP and MIPeDP have been prepared and successfully labeled with ^99m^Tc in a high labeling yield and good *in vitro* stability. Compared with ^99m^Tc-MIDP and ^99m^Tc-IPrDP, the radiolabeled complex ^99m^Tc-MIPrDP shows higher selective uptake in the skeletal system and rapider clearance from soft tissues. The results indicate that ^99m^Tc-MIPrDP is a better bone-imaging agent among our designed homologous radiotracers, and it is worthy of further preclinical investigation (such as SPECT imaging studies) to determine if it is better for bone scintigraphy. This work will be continued later.

## Figures and Tables

**Table 1:** Octanol-water partition coefficient.

Constituent	pH = 7.0	pH = 7.4
^99m^Tc-MIPrDP	−1.89 ± 0.05	−1.71 ± 0.12
^99m^Tc-MIBDP	−1.93 ± 0.09	−1.77 ± 0.05
^99m^Tc-MIPeDP	−2.10 ± 0.07	−1.89 ± 0.07

**Table 2:** Pharmacokinetic parameters of the **5a-5c** in mice (mean ± SD, n = 5, %ID/g).

Parameters	^99m^Tc-MIPrDP	^99m^Tc-MIBDP	^99m^Tc-MIPeDP
K_12_ (min^−1^)	0.0266	0.0286	0.0873
K_21_ (min^−1^)	0.0209	0.0087	0.0493
K_e_ (min^−1^)	0.0193	0.0221	0.0369
CL (%ID/g/min)	2.5887	2.9035	3.8432
T_1/2α_ (min)	11.519	12.369	4.269
T_1/2β_ (min)	102.78	202.12	61.64
AUC (%ID/g * min)	142.92	127.43	96.21

**Table 3:** Biodistribution of ^99m^Tc-MIDP and **5a-5c** in mice (mean ± SD, n = 5, %ID/g).

Tissue	Time after injection					
	5 min	15 min	30 min	60 min	120 min	240 min
^99m^Tc-MIDP [11]						
Heart	3.03 ± 0.18	1.23 ± 0.05	0.84 ± 0.01	0.54 ± 0.02	0.31 ± 0.02	0.20 ± 0.01
Liver	1.52 ± 0.03	0.68 ± 0.02	0.56 ± 0.03	0.33 ± 0.03	0.25 ± 0.01	0.16 ± 0.01
Spleen	1.73 ± 0.01	0.60 ± 0.01	0.40 ± 0.02	0.32 ± 0.04	0.32 ± 0.01	0.21 ± 0.02
Kidney	19.7 ± 1.22	12.1 ± 2.11	8.42 ± 0.22	7.82 ± 0.88	4.54 ± 0.52	4.14 ± 0.67
Muscle	1.65 ± 0.34	0.66 ± 0.02	0.34 ± 0.01	0.28 ± 0.01	0.23 ± 0.02	0.26 ± 0.03
Blood	8.54 ± 0.92	3.90 ± 0.11	1.35 ± 0.22	0.63 ± 0.02	0.30 ± 0.01	0.15 ± 0.01
All bone	11.1 ± 0.08	12.8 ± 0.23	13.2 ± 1.11	11.6 ± 1.22	14.2 ± 0.45	14.2 ± 0.23
^99m^Tc-MIPrDP						
Heart	1.41 ± 0.03	0.71 ± 0.07	0.53 ± 0.02	0.30 ± 0.01	0.12 ± 0.01	0.07 ± 0.00
Liver	1.17 ± 0.03	0.87 ± 0.06	1.35 ± 0.09	1.15 ± 0.02	0.32 ± 0.05	0.16 ± 0.00
Spleen	0.86 ± 0.03	0.59 ± 0.03	0.64 ± 0.01	0.38 ± 0.02	0.14 ± 0.00	0.07 ± 0.00
Kidney	8.35 ± 0.11	4.58 ± 0.19	3.44 ± 0.23	2.54 ± 0.05	3.21 ± 0.50	2.78 ± 0.09
Muscle	0.86 ± 0.02	0.58 ± 0.11	0.34 ± 0.03	0.19 ± 0.00	0.10 ± 0.01	0.05 ± 0.00
Blood	5.00 ± 0.02	2.69 ± 0.19	1.54 ± 0.12	0.74 ± 0.05	0.13 ± 0.01	0.08 ± 0.00
All bone	6.43 ± 0.40	10.5 ± 0.12	10.2 ± 0.56	12.2 ± 0.05	19.6 ± 0.87	11.5 ± 0.27
^99m^Tc-MIBDP						
Heart	1.43 ± 0.04	1.02 ± 0.08	0.63 ± 0.03	0.36 ± 0.02	0.16 ± 0.00	0.11 ± 0.01
Liver	1.85 ± 0.08	2.96 ± 0.07	3.59 ± 0.25	1.79 ± 0.12	1.14 ± 0.04	0.81 ± 0.01
Spleen	1.12 ± 0.03	1.05 ± 0.04	0.95 ± 0.03	0.52 ± 0.03	0.41 ± 0.03	0.29 ± 0.01
Kidney	8.36 ± 0.28	6.53 ± 0.33	5.62 ± 0.46	5.21 ± 0.40	4.45 ± 0.22	5.78 ± 0.20
Muscle	0.87 ± 0.08	0.55 ± 0.11	0.41 ± 0.03	0.15 ± 0.00	0.08 ± 0.00	0.05 ± 0.00
Blood	3.79 ± 0.07	1.54 ± 0.06	0.81 ± 0.00	0.35 ± 0.02	0.19 ± 0.00	0.09 ± 0.00
All bone	6.56 ± 0.20	11.1 ± 1.45	12.9 ± 0.42	17.1 ± 1.08	11.2 ± 0.13	16.8 ± 0.88
^99m^Tc-MIPeDP						
Heart	1.83 ± 0.05	0.99 ± 0.03	0.52 ± 0.02	0.39 ± 0.02	0.32 ± 0.03	0.16 ± 0.00
Liver	1.89 ± 0.04	5.31 ± 0.33	4.58 ± 0.16	4.20 ± 0.13	2.25 ± 0.24	0.90 ± 0.01
Spleen	1.07 ± 0.04	1.80 ± 0.14	1.45 ± 0.04	1.27 ± 0.03	0.67 ± 0.02	0.28 ± 0.02
Kidney	8.15 ± 0.26	7.05 ± 0.24	6.16 ± 0.16	5.31 ± 0.08	4.80 ± 0.14	4.06 ± 0.03
Muscle	1.08 ± 0.06	0.53 ± 0.05	0.37 ± 0.04	0.17 ± 0.01	0.11 ± 0.01	0.08 ± 0.01
Blood	3.40 ± 0.14	1.18 ± 0.08	0.91 ± 0.01	0.53 ± 0.01	0.18 ± 0.00	0.12 ± 0.01
All bone	7.22 ± 0.33	11.3 ± 1.76	11.4 ± 0.05	12.6 ± 0.13	17.6 ± 0.42	11.4 ± 0.31
